# An Increased Genotoxic Risk in Lymphocytes from Phototherapy-Treated Hyperbilirubinemic Neonates

**DOI:** 10.18869/acadpub.ibj.21.3.182

**Published:** 2017-05

**Authors:** Seyed Alireza Mesbah-Namin, Maryam Shahidi, Maryam Nakhshab

**Affiliations:** 1 Department of Clinical Biochemistry, Faculty of Medical Sciences, Tarbiat Modares University, Tehran, Iran; 2 Department of Biochemistry and Biophysics, Faculty of Medicine, Mazandaran University of Medical Sciences, Mazandaran, Sari, Iran; 3 Department of Pediatrics, Faculty of Medicine, Mazandaran University of Medical Sciences, Mazandaran, Sari, Iran

**Keywords:** Phototherapy, Hyperbilirubinemic, Neonates, Apoptosis

## Abstract

**Background:**

Phototherapy is believed to be a safe method for the management of hyperbilirubinemia. However, there are some controversial issues regarding the genotoxic effects of phototherapy on DNA. The aim of this study was to investigate morphologically both phototherapy-induced DNA double-strand breaks (DSBs) and apoptosis in lymphocytes derived from jaundiced and non-jaundiced neonates.

**Methods:**

Newborns were divided into three groups, including phototherapy-treated (PT, n=30) jaundiced newborns with total serum bilirubin (TSB) levels >15 mg/dl, non-treated jaundiced newborns (C^+^, n=27), as positive, as well as healthy negative (C^-^, n=30) controls with TSB levels ranging from 10 and 15 mg/dl and less than 5 mg/dl, respectively. Lymphocytes were isolated from whole blood samples by Ficoll-isopaque density gradient centrifugation and then assessed for DNA damage and apoptosis before and 24 hours after incubation at 37°C in 5% CO_2_ using the neutral comet assay.

**Results:**

DSB levels were significantly much higher in the PT group compared to the controls before incubation but decreased remarkably after the incubation period. As expected, no statistical differences were found between the two control groups before and after incubations. The frequency of apoptotic cells showed no significant differences among all the three groups before incubation; however, it was significantly increased in the PT group after incubation.

**Conclusion:**

It seems that phototherapy in jaundiced infants is able not only to induce apoptosis in newborn lymphocytes but also to affect indirectly DNA integrity.

## INTRODUCTION

Neonatal jaundice is a condition in which the serum level of unconjugated bilirubin increases during the first week of life. Approximately 60% of term and 80% of preterm newborns suffer from neonatal jaundice. In some cases, high bilirubin levels appear to have harmful effects on neural tissue. In this light, the principal aim of hyperbilirubinemia treatment is to reduce the high level of serum bilirubin, otherwise it may lead to kernicterus, which is also known as chronic bilirubin encephalopathy[1].

Phototherapy has been used as the best treatment modality to prevent neurotoxic effects of unconjugated bilirubin on newborn infants for over 50 years[2]. Phototherapy is believed to be a safe method for managing hyperbilirubinemia; however, there are some concerns regarding its genotoxic effects.

A variety of *in vivo* studies carried out on full-term jaundiced neonates have found no obvious differences in DNA damage between phototherapy-treated neonates and healthy controls[3,4]. In contrast, some studies have reported that increased DNA damage occurs after phototherapy in peripheral blood lymphocytes from jaundiced neonates[5-11]. However, *in vivo* follow-up studies of full-term neonates from birth to childhood[9,12] and an *in vitro* study on normal human fibroblasts exposed to visible light[13] have demonstrated that phototherapy has non-permanent adverse effects on DNA. Moreover, in long-term follow-up investigations of infants receiving phototherapy, no significant changes have been reported in growth, neuro-developmental status, and childhood leukemia[14,15].

Cells are generally equipped with DNA repair mechanisms to decrease the levels of DNA damage[16]. However, neonates have been found to have lower DNA repair and antioxidant capacities compared with adults[17,18]. Apoptosis is one of the cellular strategies to eliminate cells with high levels of unrepaired DNA damage[16]. In fact, low levels of DNA damage activate repair processes, while high levels of DNA damage (or residual DNA damage) induce apoptosis[19]. Mild genotoxic stimuli, such as low-energy radiations, induce cell apoptosis, while strong stimuli lead to cell necrosis[20].

The genotoxic effects of hyperbilirubinemia and/or phototherapy have previously been investigated using different methods such as alkaline comet assay, sister chromatid exchange (SCE), and micronucleus assay[3,5-11]. However, this is the first morphological study evaluating jaundice- and phototherapy-induced DNA double stand breaks (DSBs) and apoptosis in circulating lymphocytes from hyperbilirubinemic neonates using the neutral comet assay, a well-established and an efficient method to detect such damages in the cellular level[21,22]. Moreover, this is the first investigation to examine DNA damage and measure the number of apoptotic cells before and 24 hours after incubation, which is necessary to evaluate the exact amount of induced apoptosis.

## MATERILS AND METHODS

### Subjects

The present study included 87 newborns (3-10 days old) with gestation age of ≥35 weeks. Newborns were divided into three groups based on the total serum bilirubin (TSB) levels upon admission and the need for phototherapy. The phototherapy-treated group (PT, n=30, 16 males and 14 females) consisted of newborns with TSB levels more than 15 mg/dl and received phototherapy for 20-24 hours. The criteria used for phototherapy were similar to those previously described by the American Academy of Pediatrics[2]. The jaundiced infants, who did not receive any phototherapy, were served as a control group (C^+^, n=27, 12 males and 15 females) consisting of newborns whose TSB levels were between 10 and 15 mg/dl at the blood sampling time. The healthy negative control group (C^-^, n=30, 13 males and 17 females) was composed of newborns whose TSB levels were less than 5 mg/dl.

Certain groups were excluded from the study, including premature and postmature newborns, as well as neonates with jaundice necessitating exchange transfusion, birth asphyxia, infectious diseases, hemolytic anemia, and a history of maternal diabetes. Parents signed a written informed consent form before performing any procedure. All cases were selected from the Neonatal Intensive Care Unit in the Nimeh Shaban Hospital, Sari, Iran. The study was reviewed and approved by the Ehics Committee of the Medical Faculty of the Mazandaran University, Sari, Iran.

### Phototherapy

Phototherapy was introduced using a standard phototherapy unit (XLZ–Ningbo David Medical Device Co. China) with 11 special blue-fluorescent tubes (Philips, TL 20W/52, Germany), producing radiation with wavelengths of 400 to 500 nm at 1500 μW/cm^2^. Irradiance was measured at the skin surface of the PT group by an illuminance meter (Hagner S3, Hagner Co., Sweden). The eyes and perineal area of the neonates were masked with the black covers, while the rest of the body was exposed directly to the light. The babies showing decreased TBS to the normal range within the first 24 hours after birth were excluded from the study.

### Whole blood samples

Whole blood samples, obtained from the C^-^ and C^+^ groups at the time of admission and from the PT group approximately 24 hours after the initiation of phototherapy, were collected in heparinized tubes. Mononuclear cells were isolated from heparinized blood samples by Ficoll-hypaque (supplied by Blood Transfusion Organization of Iran, Tehran) centrifugation at 700 ×g at 20°C for 20 min. The cells were subsequently washed with PBS, resuspended in RPMI-1640 medium (Gibco, BRL, Long Island, NY, USA), and supplemented with 20% fetal calf serum (Gibco, BRL) for 24 hours. All samples were assessed for DNA damage and apoptosis at two time intervals, immediately after blood sampling and 24 hours after the incubation of cells in 5% CO_2_ at 37°C. Cryopreserved lymphocytes from a healthy individual, as an internal standard, were analyzed at two different experimental times.

### Slide preparation and neutral comet assay

The modified neutral comet assay was used to assess apoptosis and DNA damage[20]. Briefly, the samples were centrifuged at 2500 ×g at room temperature for 5 min, the supernatants were removed, and 10^5^ cells were mixed with 140 μl of 0.75% low melting point agarose (Fermentas, Lithuania) in PBS. The resulting suspension (70 µl) was layered on the top of each window of the frosted glass microscope slides (Sotooneh Co., Sari, Iran) pre-coated with a supporting layer of 1% normal melting point agarose (Fermentas, Lithuania) in distilled water. Next, it was covered with coverslips and kept at 4°C for about 5 min to solidify the gel. The coverslips were removed, and the slides were soaked in freshly-prepared lysing solution (2.5 M NaCl, 0.1 M EDTA, 10 mM Tris-base, 1% N-lauryl sarcosine, 1% Triton X-100, and 10% dimethyl sulfoxide, all supplied from Merck Company, Germany) in the dark at 4°C for 30 min. This process removes DNA-bound proteins, making movement of DNA fragments easier through an electric field. After the lysis step, the slides were washed three times in electrophoresis buffer consisting of 90 mM Tris-base, 90 mM boric acid, and 2.5 mM Na_2_EDTA (Merck, Germany), pH 8.3-8.4. The slides were then transferred into a submarine horizontal electrophoresis chamber, containing a fresh electrophoresis buffer. Electrophoresis was performed at 20 volts (0.5 V/cm) and 8 mA for 15 min. Afterwards, the slides were washed with distilled water for 5 min and fixed in ethanol at room temperature for additional 5 min. The air-dried slides were stained with ethidium bromide (20 mg/ml) and covered with coverslips prior to analysis.

### Measurement of lymphocyte DNA damage and apoptosis

**Fig. 1:**
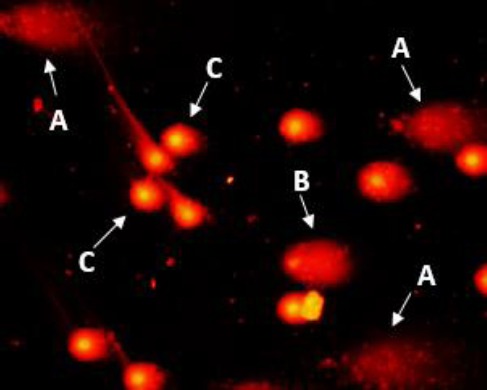
Photomicrograph of apoptotic, highly damaged and normal peripheral blood lymphocytes. A, B and C represent apoptotic, highly-damaged, and normal cells, respectively.

Cells were analyzed by using a fluorescent microscope (E-800, Nikon, Japan) equipped with an excitation filter (510–550 nm) and a barrier filter (590 nm) at 200× magnification. Figure 1 represents a typical photomicrograph of normal cells, comets, and apoptotic cells. To assess the DNA damage, images acquired from 100 randomly-selected cells were analyzed from each coded slide. The comets were analyzed by visual classification as described by Jaloszynski *et al*.[23]. Damage was assigned to five classes (0–4) based on the visual aspect of the comets, considering the extent of DNA migration, - according to the criteria established by Kobayashi *et al*.[24]. Comets with a bright head and no tail were classified as class 0 (cells with no DNA migration), while those with a small head and a long diffuse tail were classified as class 4 (severely-damaged cells). Comets with intermediate appearance were also categorized into classes 1, 2, and, 3. Damage scores were calculated based on the following equation[23], ranging from 0 to 400 arbitrary units (AU), which correspond to the situations ranging from undamaged to extremely damaged comets:

DD (AU) = (0n_0_ + 1n_1_ + 2n_2_ + 3n_3_ + 4n_4_)/(Σn /100)

where DD (AU) is DNA damage score, n_1_–n_4_, the number of class 0-4 comets, and Σn, the total number of scored comets. Coefficients 0-4 were considered as weighting factors for each comet class. Atypical comets exhibiting small or non-existent head and diffuse tails, referred to as ‘hedgehogs’ or ‘clouds’, were considered as apoptotic cells. Therefore, an increase in comet tail distribution was used to evaluate the apoptosis value[25]. A total number of 500 cells were randomly analyzed for each sample.

### Statistical analysis

All statistical analyses were carried out using GraphPad Prism software, version 6. The chi-square test was used to analyze categorical variables. A Wilcoxon’s matched-pairs signed rank test was performed to assess any differences between before and after incubation (0 and 24 h). The non-parametric Kruskal-Wallis test was used to compare differences between the average DNA damage intensity and the frequency of apoptotic cells among the groups. In addition, the Mann-Whitney U-test was applied to compare differences between the two groups. Data were expressed as the mean±SD, and *P*<0.05 was considered to be statistically significant.

## RESULTS

In the present study, the neonates were divided into the three groups based on TSB levels at the time of admission and the need for phototherapy. No statistically significant differences were found in demographic data such as sex, gestational week of delivery, birth weight, as well as admission age and weight, among the three groups (*P*>0.05, Table 1). TSB levels were significantly different among all the three groups at admission (*P*<0.001, Table 1). In the phototherapy group, a decrease in TSB levels was detected 24 hours after the initiation of phototherapy from 19.1±2.83 mg/dl before phototherapy to 13.50±2.58 mg/dl 24 hours after phototherapy. However, no statistical significances were observed 24 hours after phototherapy in TSB levels between phototherapy and C^+^ groups (12.44±1.6 mg/dl) (*P*>0.05, Table 1).

**Table 1:** Baseline demographic data of newborns in the study

Demograghic data	Non-jaundiced control (n=30) (mean ± SD)	Jaundiced control (n=27) (mean ± SD)	Phototherapy (n=30) (mean ± SD)	*P* value^a^
Males/females*	13/17	12/15	16/14	0.6900
Gestational week**	37.3±1.2	37.8±1.3	37.5±1.2	0.9704
Birth weight(g)**	3400±410	3120±558	3204±678	0.3223
Admission weight (g)**	3324±386	3093±488	3146±653	0.2173
Admission age (h)**	96.5±14	105.92±9.7^b^	114±19^b^	0.0001
TSB level at admission (mg/dl)**	4.14±1	12.44±1.6	19.1±2.83	0.0001
TSB level at sampling time (mg/dl)**	4.14±1	12.44±1.6^b^	13.50±2.58^b^	0.0001

a *P* value represents the comparison between all groups;

b there is no difference between phototherapy and jaundiced control groups (*t*-test). Newborns in the phototherapy group were sampled for the study 24 hours after phototherapy. TSB, total serum bilirubin (mg/dl); SD, standard deviation;

* Chi-square test;

** Kruskal-Wallis test

**Fig. 2:**
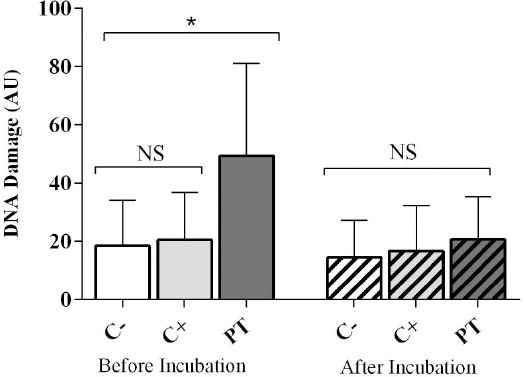
DNA damage levels before and after incubation in all three groups. DNA damage is based on arbitrary unit (AU). *Statistically significant. NS, not significant

To investigate the levels of DNA damage and apoptosis, we assessed blood samples at two time points: 1) immediately after blood sampling for the evaluation of DNA damage at time 0 (DD_0_) and apoptosis at time 0 (Ap_0_) and 2) 24 hours after incubation in 5% CO_2_ at 37ºC for the measurement of DNA damage and apoptosis at time 24 (DD_24_ and Ap_24_). The 24-h incubation time would allow the repair process and apoptotic responses to be completed. Immediately after blood sampling, the level of DNA damage (DD_0_) was found to be remarkably higher in the phototherapy group compared to C^+^ and C^-^ groups (*P*<0.0001, Tables 2 and 3, Fig. 2). Twenty-four hours after incubation, DNA damage dramatically decreased in the phototherapy group, indicating significant differences between DNA damage at time 0 and 24 of incubation (DD_0_ and DD_24_) (*P*<0.0001, Tables 2 and 3, Fig. 2). However, DNA damage at time 24 (DD_24_) measurement failed to show any noticeable differences among all the groups (*P*>0.05, Tables 2 and 3, Fig. 2). In the C^-^ and C^+^ groups, DNA damage failed to document any statistically significant differences before and after incubation (*P*>0.05, Tables 2 and 3, Fig. 2).

**Table 2:** Results related to the DNA damage (DD) and Apoptosis (Ap) of cells before and 24 hours after incubation in each study group

Groups	DNA Damage			Apoptosis		
	
	DD_0_ (mean±SD)	DD_24_ (mean±SD)	*P* value	Ap_0_ (mean±SD)	Ap_24_ (mean±SD)	*P* value
PT	49±31	20±15	=0.0002*	1.8±2.8	18.3±27.8	<0.0001*
C^+^	20±16	17±15	>0.25	1.3±1.1	1.6±1.4	>0.20
C^-^	18±15	14±13	>0.15	1.4±1.3	2±1.7	>0.03

DD_0_ and Ap_0_ indicate before, and DD_24_ and Ap_24_ represent 24 hours after incubation at 37ºC and 5% CO_2_. DNA damage and apoptosis are based on arbitrary unit (AU) and the number of apoptotic cells/500 cells, respectively. Standard deviation (SD).

* Statistically significant

**Table 3:** Results related to DNA damage (DD) and Apoptosis (Ap) of cells before and 24 hours after incubation among all three study groups.

Compared Study Groups	Before incubation		After incubation	
	
	DD_0_	Ap_0_	DD_24_	Ap_24_
PT vs. C^+^	*P*<0.0001*	*P*>0.05	*P*>0.05	*P*<0.005*
PT vs. C^-^	*P*<0.0001*	*P*>0.05	*P*>0.05	*P*<0.01*
C+ vs. C^-^	*P*>0.05	*P*>0.05	*P*>0.05	*P*>0.05

DD_0_ and Ap_0_ represent before and DD_24_ and Ap_24_ indicate 24 hours after incubation at 37ºC and 5% CO_2_.

* Statistically significant

**Fig. 3:**
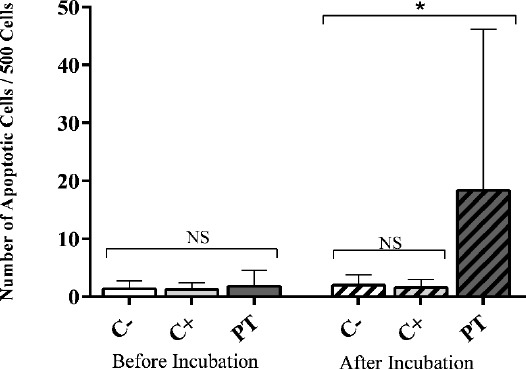
The number of apoptotic cells before and after incubation in all three groups. Apoptosis is based on the number of apoptotic cells/500 cells. *Statistically significant. NS, not significant

Considering the frequency of apoptotic cells, no statistical differences were found before incubation (Ap_0_) among all the three groups (*P*>0.05, Tables 2 and 3, Fig. 3). Interestingly, there were no statistical differences between the number of apoptotic cells before and after incubation in each C^-^ and C^+^ group (*P*>0.05, Tables 2 and 3, Fig.3). Nonetheless, a notable increase was found after incubation in the PT group (*P*<0.001) so that the number of apoptotic cells after 24 hours of incubation (Ap_24_) in the PT group was quite greater than that in C^-^ and C^+^ groups (*P*<0.01, Tables 2 and 3, Fig. 3).

## DISCUSSION

Although the pre-incubation DNA damage (DD_0_) was higher in the C^+^ group compared to the healthy newborns, the variation was not statistically significant

(*P*>0.05). Consistent with our results, Mohamed and Niazy[10], failed to find any significant differences between jaundiced and non-jaundiced control groups in terms of DNA damage and sister chromatid exchange frequency. Moreover, in a recent study[11] using the alkaline comet assay, it has been shown that hyperbilirubinemia lacks the ability to induce any genotoxic effects on DNA in jaundiced neonates. The authors attributed this incidence to the fact that bilirubin has an important physiological antioxidant role, leading to its cytoprotective feature mediated by its sacrificial oxidation[26,27]. In contrast to these findings, a variety of studies used SCE, micronucleus, and alkaline comet assays showed a correlation between high serum bilirubin levels and DNA damage in jaundiced neonates[3,7,8].

Phototherapy seems to promote the release of reactive nitrogen species, reactive oxygen species[18] and photolysis products. These cytotoxic agents are associated with the production of free oxygen radicals[28] that lead to DNA damage. In the present study, DNA damage levels were remarkably higher immediately after phototherapy (DD_0_) compared to the control groups. In supporting our findings, several studies demonstrated the genotoxic role of phototherapy in jaundiced neonates by using alkaline comet assay, SCE frequency, and micronucleus assay[5-11]. This effect is more likely due to the oxidative role of phototherapy[29,30]. In contrast to our results, Karakukcu *et al*.[3] found a reduction in DNA damage levels using the alkaline comet assay in healthy full-term hyperbilirubinemic newborns who recieved phototherapy. This damage was attributed to a decrease in bilirubin levels induced by phototherapy[3].

In the current study, the isolated lymphocytes from all the groups were incubated in 5% CO_2_ at 37°C for 24 hours. Similar to our study, a wide variety of investigators incubated cells from 24 to 72 hours to measure DNA damage and repair kinetics in different cell types. As a general rule, an incubation period provides the cells sufficient time to repair DNA damage; this event allowed several investigators to study repair kinetics during incubation[31,32]. DNA damage levels were meaningfully higher in the PT group in post-incubation period compared to controls but notably repaired after the incubation period. Several researchers investigated the residual DNA damage in terms of chromosomal breaks and found obvious differences between samples obtained before phototherapy and within 48 hours after discontinuation of phototherapy[4]. Although the repair process can theoretically decrease DNA damage after incubation[13], lower DNA repair and antioxidant capacities in neonates compared to adults should be taken into consideration[17,18]. In addition, it should be noted that the negative effects of phototherapy have previously been investigated on the antioxidant defense system in hyperbilirubinemic neonates[29].

In addition to the measurement of DNA damage, the current study has focused on the apoptotic response following DNA damage induction. The frequency of apoptotic cells was assessed before (Ap_0_) and 24 hours after incubation (Ap_24_). Cell death, another cellular strategy against DNA damage, can explain the decrease in the DNA damage level. Based on complex enzymatic reactions, apoptosis, necrosis, and autophagy are different types of cell death[16]. It is important to note that the apoptosis process might be initiated during the phototherapy; however, it needs an incubation of 24 hours to be distinguishable morphologically[32]. Other investigators also evaluated apoptosis after an incubation period of 24 hours using the neutral comet assay[33,34].

In our study, the number of apoptotic cells before the incubation (Ap_0_) showed no significant differences between all three groups. Furthermore, there was no correlation between hyperbilirubinemia and the number of apoptotic cells. On the other hand, the measurement of *P53* in jaundiced babies and control groups showed that hyperbilirubinemia is unable to induce apoptotic responses[11]. However, the apoptosis-inducing effect of jaundice was verified in a study carried out by El-Abdin *et al*.[8], in which plasma *Bcl*2 and *BAX* genes expression was measured in hyperbilirubinemic newborns prior to phototherapy[8].

Additionally, the apoptosis-inducing effect of phototherapy was confirmed when the *P53* level and plasma *Bcl*2 and *BAX* genes expression were evaluated in phototherapy-treated jaundiced newborns immediately prior to phototherapy discontinuation[8,11]. In contrast, no increased frequency of apoptotic cells was observed immediately after phototherapy (Ap_0_) in the PT group compared to that in controls. The critical difference between our results and two above-mentioned studies in jaundiced neonates can be justified by different phases of apoptosis process and duration. Apoptosis-induced morphological changes, which are mainly investigated in the present study, are the last step of apoptosis[35]. The *P53* level as well as plasma *Bcl-2* and *BAX* genes expression are considered to be signals affecting apoptotic responses, and any changes in their levels are associated with apoptosis[16]. Moreover, the duration of apoptosis may vary from 12 to 24 hours, depending on the stimulus and cell type[36]. Therefore, we attempted to measure the frequency of apoptotic cells 24 hours after incubation, allowing the apoptosis processes to be completed. However, the studied neonates were not followed up to determine long-term side effects of phototherapy. In addition, since blood samples were taken from neonates at only time point, we were unable to compare study parameters before and after phototherapy in the PT group.

Based on our results, the C+ group displayed no significant change in Ap_24_, as compared to Ap_0_. This is presumably due to the inability of hyperbilirubinemia to induce significant DNA damage, which in turn results in no apoptotic induction[11]. The high amount of Ap_24_ in the PT group after incubation highlights the DNA damage inducing potential of phototherapy.

The apoptosis-inducing activity of phototherapy and jaundice has previously been investigated[8,11]. These studies have focused on some markers initiating apoptosis responses. However, we attempted to morphologically evaluate apoptosis using the neutral comet assay. In addition, most studies investigated the effects of jaundice and/or phototherapy on DNA damage in neonates using the alkaline comet assay[3,5,6,11], which allows the detection of both single and DSBs[37]. In contrast, the present study used the neutral comet assay that has the ability to detect only DSBs, which are more important than single strand breaks to induce chromosomal aberrations[38] and apoptotic responses at the cellular level[21]. Concerning this view, our study is the first investigation to examine both DNA damage and the frequency of apoptotic cells before and 24 hours after incubation to demonstrate the safety of phototherapy in contrast to its genotoxic effects. We believe that further investigations are required to determine the repair activity during the incubation period in lymphocytes from phototherapy-treated neonates.

Taken together, it can be stated that jaundice has no negative impacts on DNA and on apoptosis induction. In contrast, phototherapy, the most commonly used treatment for jaundiced newborns, can negatively influence DNA integrity and induce genotoxic effects. Hence, more considerations are needed regarding the indications of phototherapy in hyperbilirubinemic neonates.
